# Towards malaria elimination in Mpumalanga, South Africa: a population-level mathematical modelling approach

**DOI:** 10.1186/1475-2875-13-297

**Published:** 2014-08-03

**Authors:** Sheetal P Silal, Francesca Little, Karen I Barnes, Lisa J White

**Affiliations:** 1 Department of Statistical Sciences, University of Cape Town, Cape Town, South Africa; 2 Division of Clinical Pharmacology, Department of Medicine, University of Cape Town, Cape Town, South Africa; 3 Mahidol-Oxford Tropical Medicine Research Unit, Mahidol University, Bangkok, Thailand; 4 Centre for Tropical Medicine, Nuffield Department of Clinical Medicine, Churchill Hospital, University of Oxford, Oxford, UK

## Abstract

**Background:**

Mpumalanga in South Africa is committed to eliminating malaria by 2018 and efforts are increasing beyond that necessary for malaria control. Differential Equation models may be used to study the incidence and spread of disease with an important benefit being the ability to enact exogenous change on the system to predict impact without committing any real resources. The model is a deterministic non-linear ordinary differential equation representation of the dynamics of the human population. The model is fitted to weekly data of treated cases from 2002 to 2008, and then validated with data from 2009 to 2012. Elimination-focused interventions such as the scale-up of vector control, mass drug administration, a focused mass screen and treat campaign and foreign source reduction are applied to the model to assess their potential impact on transmission.

**Results:**

Scaling up vector control by 10% and 20% resulted in substantial predicted decreases in local infections with little impact on imported infections. Mass drug administration is a high impact but short-lived intervention with predicted decreases in local infections of less that one infection per year. However, transmission reverted to pre-intervention levels within three years. Focused mass screen and treat campaigns at border-entry points are predicted to result in a knock-on decrease in local infections through a reduction in the infectious reservoir. This knock-on decrease in local infections was also predicted to be achieved through foreign source reduction. Elimination was only predicted to be possible under the scenario of zero imported infections in Mpumalanga.

**Conclusions:**

A constant influx of imported infections show that vector control alone will not be able to eliminate local malaria as it is insufficient to interrupt transmission. Both mass interventions have a large and immediate impact. Yet in countries with a large migrant population, these interventions may fail due to the reintroduction of parasites and their impact may be short-lived. While all strategies (in isolation or combined) contributed to decreasing local infections, none was predicted to decrease local infections to zero. The number of imported infections highlights the importance of reducing imported infections at source, and a regional approach to malaria elimination.

## Background

**Figure 1:**
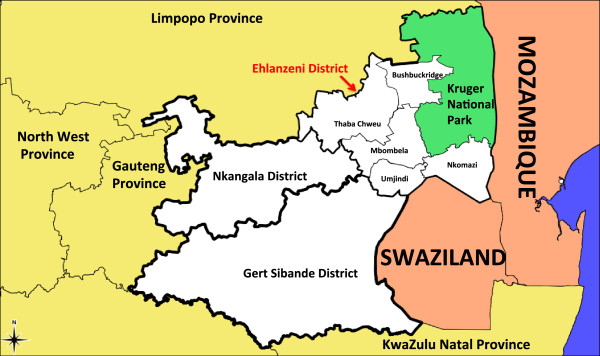
**Mpumalanga province.** A map of Mpumalanga Province in relation to Mozambique and Swaziland (Source: Mpumalanga Malaria Elimination Programme (unpublished)).

**Figure 2:**
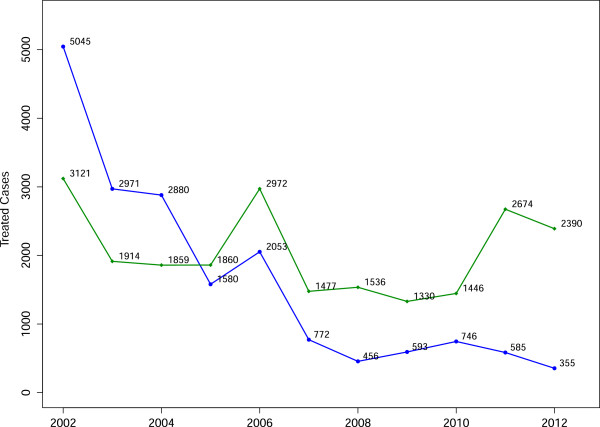
**Annual malaria incidence.** Annual incidence of locally sourced (blue) and imported (green) malaria cases that have been reported and treated at health facilities in Mpumalanga province.

Mpumalanga is one of three malaria-endemic provinces in South Africa. As South Africa is committed to eliminating malaria by 2018, efforts are increasing in each of these three provinces beyond that which was necessary for malaria control [1]. In shifting focus from control to elimination, additional activities need to be incorporated into the operational strategy as elimination may not be achieved through a “more of the same” approach [2]. Groups such as the MalERA Consultative Group on modelling, have recognized the contribution mathematical modelling can make to the elimination of malaria globally and have highlighted priority areas that modelling can inform, such as optimal resource allocation, strategies to minimize the evolution of drug and pesticide resistance, assessment of new tools to interrupt malaria transmission, assessment of combinations of such tools, the coverage targets and expected timelines needed to achieve elimination goals and the assessment of operational feasibility with respect to costs and human resource capacities [3]. While such models may be used to understand/explore the underlying system, an important benefit of modelling is the ability to enact exogenous change on the system to predict impact without committing any real resources. This is particularly important in environments with scarce competing resources. In this paper, a population-based non-linear ordinary differential equation model is used to simulate malaria transmission in Mpumalanga province to assess the potential impact of various policy interventions that may be used to achieve malaria elimination. Malaria in Mpumalanga has been documented extensively [4-10]. Sharing borders with both Mozambique and Swaziland (Figure 1), Mpumalanga experiences seasonal unstable transmission that is prone to sporadic outbreaks from the first rains in October to late May. The Ehlanzeni District on the eastern border of Mpumalanga is most affected by malaria with the number of imported cases in Mpumalanga overtaking locally sourced cases in recent years (Figure 2). Between 2002 and 2012, 41% of cases were sourced in South Africa and 54% sourced from Mozambique (the remaining 5% being sourced from other African and Asian countries). Source of infection has been determined for all cases in the province, whereby a case is classified as imported if the patient travelled to a malaria-endemic area in the past month or if there is no evidence of local transmission (vectors or cases within 500 m radius of the place of residence) [11]. The proportion of imported cases has increased from 39% in 2002 to 87% in 2012 [12]. Extensive vector control through indoor residual spraying, the implementation of artemisinin-based combination therapy policy of artensunate plus sulphadoxine-pyremethamine in 2003, followed by artemether lumefantrine (AL) in 2006 and the Lubombo Spatial Development Initiative (LSDI) are considered responsible for the decline in malaria cases and malaria deaths in the province [4]. The LSDI malaria programme was a collaborative project conceived by the Medical Research Councils of South Africa, Swaziland and Mozambique to decrease malaria in the areas surrounding the Lubombo Mountains [13]. With well-functioning programmes in place in both South Africa and Swaziland, intervention took place primarily in Maputo province and Gaza Provinces in Mozambique. The programme was terminated early in September 2010 and the resultant reduced IRS in Maputo thereafter coincides with the increase observed in malaria incidence in Maputo from 2011.

Differential Equation or compartment models, have been used in the past to study the incidence and spread of disease, and the impact of interventions such as drug treatment and parasite control [14]. Compartment models and their applications in malaria in particular, have a history that spans more than 100 years [15]. Applications of mathematical modelling in Mpumalanga include a climate-based fuzzy distribution model of malaria transmission in sub-Saharan Africa (including a region containing Mpumalanga) [16]. Coleman *et al*. used the SaTScan methodology in Mpumalanga to detect local malaria clusters to guide the provincial control programme [17], and Montosi *et al*. considered soil-water content as a driver of malaria incidence; applying both an ecohydrological model and a transfer function model to incidence data in three South African provinces (including Mpumalanga) [18]. The model presented in this paper is used to assess the impact of proposed policy interventions in Mpumalanga. This is the first study designed for this purpose in Mpumalanga and the first to do so since the call for malaria elimination in South Africa. A deterministic population-based non-linear ordinary differential equation model fitted to the Mpumalanga malaria data, is used to predict the impact of the following interventions (alone and in combination): scale-up of vector control, mass drug administration (MDA), a focused mass screen and treat campaign (MSAT) and foreign source reduction.

## Methods

### Transmission model

**Figure 3:**
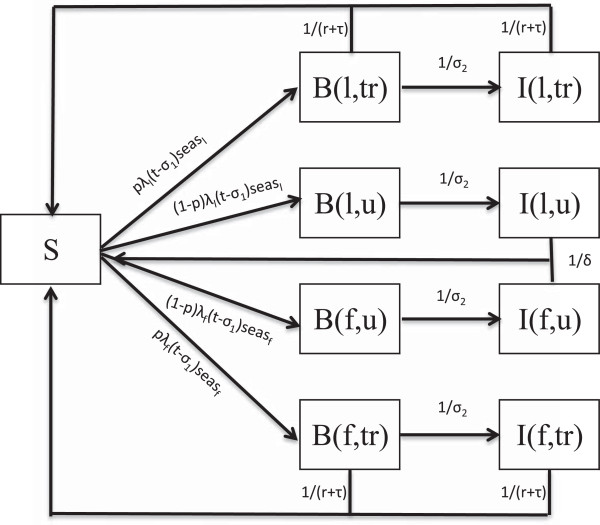
**Model flowchart.** Flowchart underlying the population level ordinary non-linear differential equation model of malaria transmission. (l - locally sourced infections, f - imported infections, u - untreated infections, tr- treated infections).

The model is a deterministic ordinary non-linear differential equation representation of the dynamics of the human population. In the model, the population is divided into nine compartments: the susceptible population (S), the asexual blood stage only (B_*l*_ and B_*f*_) for locally and imported infections respectively and the infectious gametocyte stage (I_*l*_ and I_*f*_) for locally and imported infections respectively (Figure 3 with parameter descriptions in Table 1). The blood stage and infectious stage compartments are further stratified according to whether the infection is treated or not. The liver stage of the infection is incorporated as a delay in the flow between the susceptible and blood Stage compartments. As this is a low transmission environment, immunity and super-infection are rare and are excluded from this model. While the seasonal nature of transmission is incorporated in the model, the mosquito population is not modelled directly as it is assumed that the mosquito dynamics operate on a faster time-scale than the human dynamics and as such the mosquito population can be considered to be at equilibrium with respect to changes in the human population [19]. A full description of the model is presented in Additional file 1.

**Table 1:** Parameter table

**Parameter**	**Description**	**Value**	**Source**
N	Population size	4 ×10^6^	[20]
*μ*	Mortality Rate	$\frac{105}{10000}$	[21]
*δ*	Natural recovery period	26 weeks	[22-24]
*σ*_1_	Period between liver stage and blood stage	7 days (5-10)	[25-27]
*σ*_2_	Period between blood stage and onset of gametocytemia	1 week	[23,28]
*r*	AL elimination half-life	6 days (3-6)	[29]
*τ*	Time to seek treatment	1/2 week	Expert opinion
*p*	Proportion that receive treatment	0.95	[30,31]
*seas*_*l*_	Seasonal forcing function for locally sourced cases	Derived from data	[12]
*seas*_*f*_	Seasonal forcing function for imported cases	Derived from data	[12]
*β*_*l*_	Annual number of mosquito bites per person x proportion of bites testing positive for sporozoites	39.170 (38.894, 39.448)	Estimated from model fitting process
*λ*_*f*_	Force of imported infections	0.002163 (0.002124, 0.002202)	Estimated from model fitting process
*λ*_*l*_	Force of locally sourced infections	$(1-\text{vc}[t])\beta_{l}\times \frac{I_{l,u}+I_{l,\text{tr}}+I_{f,u}+I_{f,\text{tr}}}{N}$	
*vc*[*t*]	*vccov*×*vceff*		
*vccov*	Vector Control Coverage	0.22-0.90	Derived from data
*vceff*	Effectiveness of vector control	0.9060 (0.8884, 0.9212)	Estimated from model fitting process

Table providing the values, descriptions and sources of the parameters driving the mathematical model of transmission.

### Data fitting

The model is fitted to weekly incidence data of treated cases from 2002 to 2008, and then validated with data from 2009 to 2012. Ethical approval for use of the data was obtained from the University of Cape Town Human Research Ethics Committee and the Mpumalanga Department of Health. The seasonal forcing functions (functions that determine the seasonal behaviour of transmission in the area) for local and imported cases are derived from the data. Silal *et al*. describes in detail the characteristic triple peaked pattern in the incidence data with peaks in the malaria season occurring in September/October, December/January and April/May. While locally sourced infections exhibit this triple-peaked pattern, imported infections occur mainly in the second two peaks of the malaria season [12]. The two seasonal forcing functions were derived using the “Seasonal decomposition of Time series by LOESS” (STL) methods for extracting time series components [32]. In order for the data-fitting process to be plausible, interventions that were implemented between 2002 and 2008 were included in the model, namely, ACT drug therapy and Indoor Residual Spraying (IRS). Ngomane and de Jager outline in detail the IRS procedure and physical structures sprayed in the province between 2001 and 2009 [5].

The model is run from 1990 to reach a steady state before being fitted to data from 2002. The model output (local and imported treated cases) are fitted to the data from 2002 to 2008 using the maximum likelihood approach by treating the model output as the rate *λ* of the Poisson distribution. The parameters *β*_*l*_, *veff* and *λ*_*f*_ are estimated through the data fitting process with initial values sampled from a Latin square framework. The model with the estimated parameter values is then run for a further three years to be further validated by comparison to data between 2009 and 2012. The impact of routine drug therapy and IRS implemented between 2009 and 2012 is also included in the model. A full description of IRS and the data-fitting method are presented in Additional file 1.

### Assessing elimination

Elimination of a disease is a term that has had several definitions over time [33]. Currently, the World Health Organization(WHO) defines elimination generally to be “Interrupting local mosquito-borne malaria transmission in a defined geographical area, i.e. zero incidence of locally contracted cases, although imported cases will continue to occur. Continued intervention measures are required” [34]. The framework of deterministic differential equation models are such that compartments may approach zero but will never actually decrease to zero; hence it is technically impossible for the model to predict zero incidence of locally contracted cases. It is then necessary to set a threshold below which the number of locally contracted cases is deemed equivalent to zero. This approach has been used in several papers. For example, defined elimination as having been achieved when parasite prevalence is reduced to 0.0001% and the rate of change in parasite prevalence thereafter is negative [22]. Maude *et al.* defined elimination to be achieved when there is fewer than one malaria parasitaemic individual in the population [35]. The threshold for elimination that is adopted in this paper is less than one locally sourced malaria infection per year in the population and the rate of change of locally sourced infections is negative thereafter. When the model predicts that the number of locally contracted cases is below this threshold and the rate of change of locally sourced infections is negative thereafter, then elimination is predicted to occur.

## Results

The results of the model fitting and validation are presented first before evaluating the predicted impact of the elimination-focused interventions.

### Model fitting and validation

**Figure 4:**
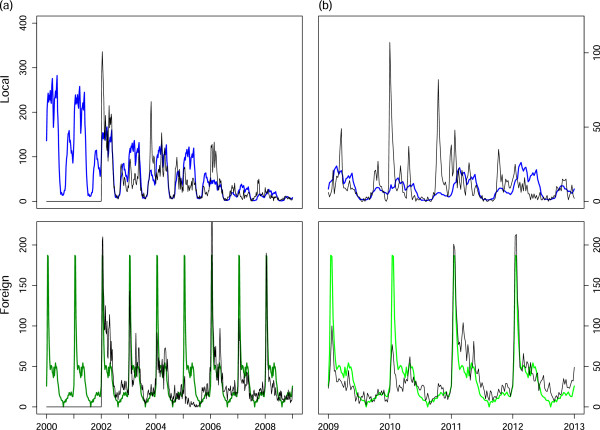
**Model fitting and validation to treated case data. (a)** Model fitting results to locally sourced (blue) and imported (green) reported treated malaria cases and **(b)** Model validation results to locally sourced (blue) and imported (green) reported treated malaria cases from 2009 to 2012.

The parameters driving the model and their 95% confidence intervals estimated through data-fitting procedures are presented in Table 1. A parameter that is usually unknown and estimated from the data is the proportion of cases treated (*p*). Case data usually includes cases that have been treated, comprising patients presenting themselves at a health facility (passive case detection), or those cases that have been detected actively. There is often no indication of the number of infections that have remained untreated, and hence there is no/little data from which to derive *p*. Castillo-Riquelme *et al*. conducted household surveys in Mozambique and South Africa between 2001 and 2002 to evaluate treatment-seeking behaviour for malaria-related events [30]. It was found that in the Tonga sub-district of Mpumalanga, all of the 457 people with recent cases of malaria (previous month) sought treatment, with 98.5% of cases being treated at a public health facility. More recently, Hlongwana *et al*. conducted a study on the knowledge and practices towards malaria in Bushbuckridge Municipality in Mpumalanga in 2008 after South Africa was declared ready for malaria elimination [31]. The study revealed that 99% of respondents would seek malaria treatment (95% Confidence interval: (97.5, 99.5)%) with 82% doing so within 24 hours of the onset of symptoms. Based on these two studies, a probability of treatment of 95% is assumed in the model for the entire modelling period as it is informed by studies conducted in 2002 and 2008.Model fitting was performed using different starting values of the parameters with the optimization routine reaching the global minimum in almost all fits. The narrow confidence intervals of the parameter estimates are indicative of the stability of the estimates. Figure 4(a) shows the fit of the model to the data for the treated cases that were locally and imported while Figure 4(b) shows the application of the model to treated case data from 2009 to 2012. The model captures the timing of the season well. As seen in Figure 4(b), there is a sudden unanticipated rise in the number of imported cases in 2011 and 2012 coinciding with the end of the Lubombo Spatial Development Initiative and the model does not capture this.

### Interventions

The model is used to predict the impact of the following interventions (alone and in combination): scale-up of vector control, mass drug administration (MDA), a focused mass screen and treat campaign (MSAT) and foreign source reduction.

#### Scaling up vector control

**Figure 5:**
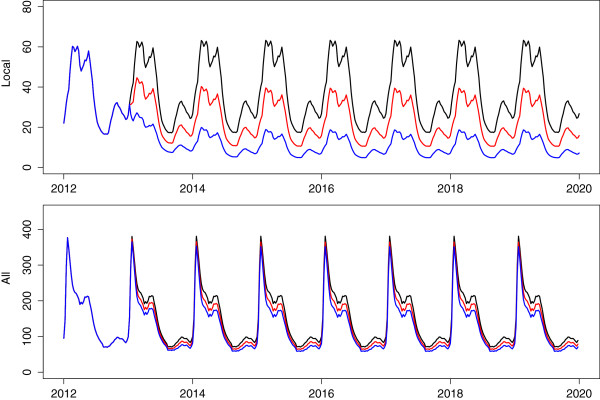
**Scale-up of vector control.** Predicted impact of a scale up of vector control that reduces transmission by a further 10% (red) and 20% (blue).

The primary exogenous vector control intervention in use currently is IRS with larviciding at specified sites. As these interventions act directly on local vectors and hence local transmission, their impact is included in the model as a percentage decrease in *β*_*l*_, the number of local human contacts with infectious mosquitoes. By 2012, the vector control activities decreased *β*_*l*_ to 30% of its initial value (Figure 5, black). Scaling up vector control (through increased IRS or larviciding for example)so as to decrease *β*_*l*_ by a further 10% (Figure 5, red) and 20% (Figure 5, blue) results in local infections decreasing substantially without any sizeable decrease in total infections. This is to be expected as the majority of infections are imported, and as such vector control may assist with onward local transmission of these imported infections but not change the number of imported infections itself. IRS already takes place on a large scale in the Ehlanzeni district [5]. Scaling up vector control by these amounts will require other vector control activities to be implemented such as larviciding. To achieve such reductions will require large-scale identification and coverage of breeding sites and even still the model does not predict the reduction to zero of local cases. Local infections will still occur because of onward transmission from imported infections.

#### Mass interventions

**Figure 6:**
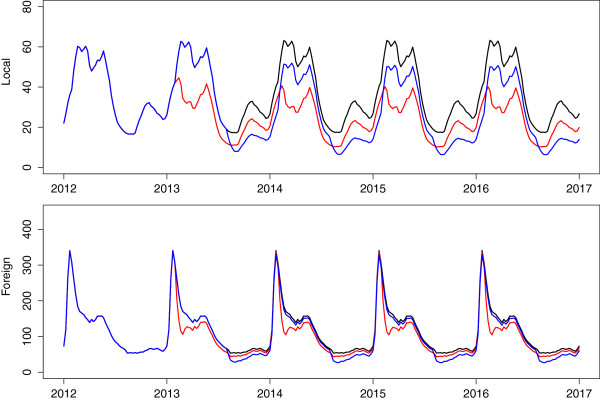
**Annual rounds of MDA.** Predicted impact of annual rounds of MDA performed at the peak (red) and trough (blue) of the season on local and imported infections.

**Figure 7:**
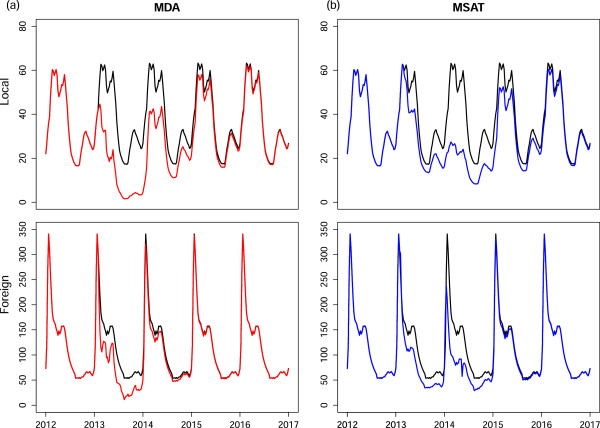
**Continuous MDA and MSAT.** Predicted impact of **(a)** six consecutive 2-monthly rounds of MDA on local and imported infections and **(b)** six months of MSAT of new imported infections at 70% coverage (border screen and treat).

One intervention that simultaneously impacts both local and imported infections is mass drug administration. Mass drug administration involves treating all individuals without prior screening to assess disease status. While MDA is aimed at the entire population of interest, it is rarely the case that every single individual will be treated and hence MDA should be modelled with a less-than 100% coverage. The choice of drug is key to the intervention as drugs that infer a long period of chemoprophylaxis may result in fewer infections after the intervention, but also expose parasites to sub-therapeutic levels of drugs which may in turn lead to the development of resistance. The most likely choices for drugs in Mpumalanga are the first-line of treatment, artemether-lumefantrine, and dihydroartemsinin plus piperaquine with Primaquine, inferring a protective period of approximately one month [36]. The timing of the MDA is also vital to its effectiveness. It may be the case that performing MDA at the trough of the season will result in fewer malaria infections and a decrease in the infectious reservoir, leading to fewer infections at the peak of the season. Figure 6 shows the predicted impact of MDA applied over a two month period annually to the whole population with 80% coverage. The timing of MDA was investigated at both the peak (Figure 6, red) and trough of the season (Figure 6, blue). It is predicted that applying MDA leads to a substantial decrease in local infections regardless of the timing of application. However, the predictions show that applying annual rounds of MDA at the peak of the season leads to substantial decreases in both local and imported infections unlike MDA at the trough of the season. A possible reason for this difference in impact is the large proportion of imported infections. Applying MDA at the season’s trough decreases imported infections then but there is no further decrease during the peak of the season as imported infections are sourced outside South Africa and so, are not impacted by the decrease in onward transmission brought about by MDA. What is also evident from Figure 6 is that annual rounds of MDA are predicted to be insufficient to bring about malaria elimination. A single round of MDA is predicted to cause an immediate decrease in infections but future transmission recovers quickly. Figure 7(a) shows the application of six consecutive two-monthly rounds of MDA to the malaria transmission model. In this scenario, local infections are predicted to decrease substantially during the MDA, reaching less than 1 local case per year (1/52 cases per week) 29 weeks after the start of the intervention and take approximately two years to recover to pre-MDA levels once the MDA has stopped. Imported infections are predicted to decrease substantially during MDA acting as a form of intermittent preventative treatment and recover immediately after the MDA cycle.

MDA is a resource-intensive process attempting to access an entire population of interest while administering a drug regardless of whether individuals have the disease or not. Mass screen and treat campaigns on the other hand treat only those that have tested positive for the disease. Figure 7(b) shows the predicted impact of administering MSAT at the border to residents of Mpumalanga who have imported infections i.e. before entering Mpumalanga. The rationale behind this intervention is that it is less resource-intensive than MDA and specifically targets imported infections before they enter the province and impact local transmission. In Figure 7b, MSAT is applied continuously in the model to new imported infections before entering Mpumalanga for six months from November to April with a coverage below 100% as many imported infections may be missed for reasons such as illegal immigration and sensitivity of the screening tools. Figure 7b also shows the substantial predicted decrease in locally sourced infections that can be achieved by treating local people who have *imported infections only* through MSAT with 70% coverage. Once again however, as soon as the intervention stops, imported infections are predicted to revert immediately to previous levels while local infections take approximately two years to reach previous levels. Lower coverage rates (<70*%*) have also been explored, with the result of even smaller decreases in locally sourced infections.

#### Combining interventions

**Figure 8:**
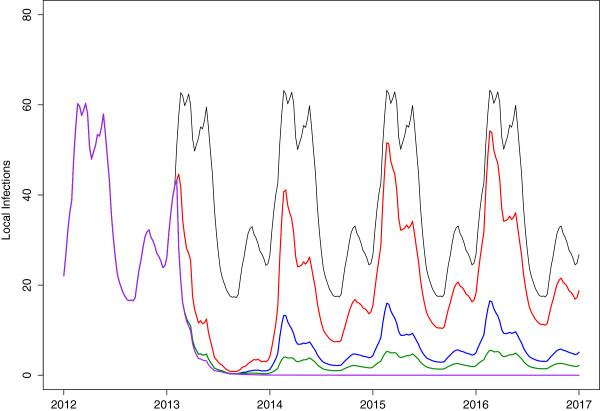
**Combination of interventions.** Predicted impact of combination of interventions on local infections: Black: No additional interventions, Red: 70% coverage of MSAT on local population with new imported infections following 6 months of continuous MDA at 80% coverage, Blue: same as red (MDA+MSAT) with increased vector control to decrease transmission by a further 20%, Green: six consecutive two-monthly rounds of MDA with increased vector control, and 70% decrease in the foreign force of infection, Purple: six consecutive two-monthly rounds of MDA with zero imported infections.

Figure 8 shows the predicted impact of a number of different combinations of interventions on locally sourced infections. The red line depicts the predicted impact of six consecutive two-monthly rounds of MDA followed immediately by MSAT (at the border on local people with new imported infections) enacted annually for six months from November to April at a 70% coverage rate. While these two mass interventions are not enough to eliminate local infections, a new substantially lower stable cycle is reached for local infections. If these interventions are supplemented with increased vector control (so as to decrease transmission by a further 20%), local infections reach very low levels with a maximum of 16 local infections per week at the peak of the season (blue). However, this resource-intensive combination of interventions is also predicted to be insufficient to achieve elimination of local malaria infections. The modelling of MSAT shows that acting on the imported infections of local people traveling back into Mpumalanga alone decreases the number of locally sourced cases. It is expected that the impact would be greater if the source of imported infections were targeted. Thus the green line is the model prediction based on foreign source reduction i.e. if the force of imported infection *λ*_*f*_ were decreased by 70% and locally six consecutive two-monthly rounds of MDA were applied and vector control was increased. Additionally, the purple line shows the predicted impact on local infections if there were no imported infections at all. In this scenario, the only other intervention that was applied was six consecutive two-monthly rounds of MDA. This combination is sufficient to eliminate local malaria in Mpumalanga. It is predicted to take only 28 weeks on average from the end of the MDA cycle to reach below one local infection per year.

## Discussion

South Africa has been employing vector control to control malaria since 1931 [37]. Reliance on vector control has been such that insecticide resistance to pyrethroids in 2000 resulted in a surge in malaria cases that could not be controlled through drug therapy alone. Consistent and large-scale IRS is considered one of the key reasons why that malaria has been so well controlled in the country and in Mpumalanga. Scaling up IRS even further through targeted larviciding of vector breeding sites may be contemplated as a strategy to achieve malaria elimination. Scaling up vector control in the mathematical model for Mpumalanga, allowed local infections to decrease to a new equilibrium but did not eliminate local infections or decrease total infections substantially. It is expected that total infections will not decrease substantially as the majority of infections in Mpumalanga are imported and local vector control will not impact the number of imported infections in the province but will work towards decreasing onward transmission of these infections. It is because of this constant influx of imported infections that vector control alone will not be able to eliminate local malaria. A “more of the same” approach does not appear to work as it is insufficient to interrupt the transmission stable cycle.

Mass drug administration is a resource intensive intervention that needs to be acted out quickly, systematically and efficiently. It is a strategy that has not found favour in recent years; one reason being that drug resistance is a feared consequence of MDA [38]. In a global environment seeking to protect the artemisinins (and their partner drugs) from drug resistance, the choice of drug for mass interventions has both global and economic importance. Economically, the drug needs to be affordable to be deployed on a large scale and globally, if resistance spreads to a point where the drug is no longer useful, the drug should be one that can be sacrificed. While the emergence of drug resistance has not been directly linked with MDA, it is possible that MDA will increase the selection pressure on the parasite population, with the possibility of losing the drug eventually [39]. In contrast, in fitting a mathematical model to trial results from Western Cambodia to assess the effects of elimination strategies and their interactions with artemisinin resistance, Maude *et al*. found that the proportion of artemisinin resistant infections increased quickly when ACT was introduced for treatment (in an area where treatment comprised non-artemisinin anti-malarial drugs and a low level of artemisinin monotherapy) and only slightly more when MDA with an ACT was implemented [35].

Both MDA and MSAT are interventions that have an immediate and large impact. Yet in countries with a large migrant population or populations with sub-optimal coverage, these interventions can fail because of the reintroduction of parasites and their impact may be short-lived [39]. Given the large proportion of imported cases in Mpumalanga, the model predicts that when a mass intervention targeting imported infections is stopped, both local and imported infection levels revert back to pre-intervention levels with a few years. This is because forces within Mpumalanga do not determine the level of imported infections; it is determined by the prevalence of malaria in the source country itself. Hence as the results of applications of MDA and MSAT in the model have shown, the large predicted impact can only be sustained if the mass interventions are applied often, and this is a resource-intensive strategy. Applying MSAT to local residents with imported infections in the model has predicted a substantial “knock on” decrease in local infections. This is expected as decreasing imported infections decreases the infectious reservoir in the province, which in turn decreases local malaria transmission. The model also predicted the substantial impact decreases in the foreign force of infection (foreign source reduction) has on local transmission. The scenario of zero imported infections was the only situation in which the model predicted that elimination of malaria could be achieved. This highlights the importance of source-reduction, monitoring imported infections and the receptivity of key areas within Mpumalanga. If the malaria vectors are present and ecological and climatic factors favour transmission in these areas, then onward transmission is probable even in the presence of good malaria control [40]. One strategy to decrease malaria transmission to zero would be to eliminate the mosquito vector population. This is highly unlikely to be feasible. As a result, even if low levels of malaria prevalence have been achieved, imported infections will augment the infectious reservoir, and since the vector remains, imported infections may lead to onward transmission to the local population and a resurgence of malaria generally [2].

In this deterministic model, a threshold was defined to determine elimination as it is mathematically impossible to achieve zero cases in a differential equation framework. As is the case with selecting any threshold, more severe thresholds could always be selected. It is of interest in this manuscript that malaria elimination could not even be predicted with the current threshold, let alone more severe thresholds. In a review on the historical and current definitions of malaria, Cohen *et al*. defines three states of malaria transmission to be controlled low-endemic malaria, elimination and controlled non-endemic malaria. This third state describes the situation where the interruption of endemic transmission has occurred but there is still malaria resulting from onward transmission from imported infections and this onward transmission is sufficiently high that elimination has not yet been achieved [33]. This implies that if all onward infection from imported infections could be prevented, elimination of malaria would follow naturally. The results of this modelling exercise suggest that Mpumalanga is in this third state of transmission as elimination of malaria is only predicted to be possible with unrealistic, resource-intensive interventions that result in a drastic reduction in imported infections. In realizing that the key to decreasing local infections further is to prevent imported infections, new approaches must be explored both nationally and regionally.

The transmission model presented in this paper is a deterministic population-level one considering only the population of Mpumalanga. While population-level models are useful to assess aggregate effects, the entire population is treated as homogenously affected by malaria transmission. Thus there is no scope to include spatial variation or heterogeneous behaviour in the transmission model or in the interventions themselves. Imported infections play an important role in transmission dynamics in Mpumalanga and human migration is modelled indirectly. Current work includes disaggregating the population into smaller groups such as administrative districts, and using a stochastic, meta-population model of transmission to model these districts while explicitly incorporating human migration between Mozambique (the main source of imported infections) and Mpumalanga. This will allow one to assess the impact of deploying interventions sequentially in the administrative districts as well as in Mozambique itself. For example, Silal *et al*. found that infections in Bushbuckridge municipality occur at higher levels at the start of the season compared to other municipalities in Mpumalanga [12]. It may be of interest then to assess if intervening in this municipality at the start of the season has a knock-on effect on malaria in the rest of the province. Spatial heterogeneity will also allow for improved modelling of interventions that target imported infections such as border control through MSAT, and source reduction in Mozambique. Future work also includes incorporating vector population dynamics in the model so that vector control activities such as indoor residual spraying and larviciding may be modelled explicitly.

## Conclusions

South Africa aims to achieve malaria elimination by 2018. This requires synchonized action in the three provinces in which malaria occurs in order to decrease local infections to zero. In the case of Mpumalanga, given the large proportion of imported cases, interventions also need to target imported infections to decrease the infectious reservoir impacting local transmission. In isolated countries, a nationally focused elimination programme may stand a better chance of success than countries with high levels of visitation from higher transmission regions [41]. This paper has used population level mathematical modelling to model transmission in Mpumalanga and test out strategies (MSAT, MDA, increased vector control and foreign source reduction) that may be used to achieve malaria elimination. While all strategies (in isolation or combined) contributed to decreasing local infections, none was able to decrease local infections to zero due mainly to the continuous stream of imported infections highlighting the importance of source reduction and a regional approach to malaria control and elimination. Disaggregating the model into smaller groups will allow for the spatial heterogeneity required to optimize elimination strategy that may lead to different results. Mathematical modelling has the potential to inform government policy to achieve malaria elimination and with effective and efficient interventions, adequate sustainable finance, local and international political commitment and an epidemiological understanding of malaria elimination, malaria elimination in Mpumalanga may be possible in the foreseeable future.

## Competing interests

The authors declare that they have no competing interests.

## Authors’ contributions

SPS wrote the paper and performed the mathematical model development and analysis. SPS and LJW conceptualised the mathematical model and analysis. FL, KIB and LJW reviewed the manuscript extensively. All authors have read and approved the manuscript.

## Supplementary Material

Additional file 1 **Mathematical Model Description.** Additional file containing model equations, description and parameter values. Click here for file

## References

[B1] National malaria elimination strategy 2011–2018 Tech. rep., South Africa National Department of Health, Pretoria, 2011

[B2] Moonen B Cohen JM Snow RW Slutsker L Drakeley C Smith DL Abeyasinghe RR Rodriguez MH Maharaj R Tanner M Targett G Operational strategies to achieve and maintain malaria elimination Lancet 2010 376 1592 603 [http://www.pubmedcentral.nih.gov/articlerender.fcgi?artid=3037542&tool=pmcentrez&rendertype=abstract] 21035841 10.1016/S0140-6736(10)61269-X PMC3037542

[B3] The malERA Consultative Group on Modeling A research agenda for malaria eradication: modeling PloS Med 2011 8 e1000403 [http://www.plosmedicine.org/article/info:doi/10.1371/journal.pmed.1000403#s4] 21283605 10.1371/journal.pmed.1000403 PMC3026697

[B4] Moonasar D Nuthulaganti T Kruger PS Mabuza A Rasiswi ES Benson FG Maharaj R Malaria control in South Africa 2000-2010: beyond MDG6 Malar J 2012 11 294 [http://www.malariajournal.com/content/11/1/294] 22913727 10.1186/1475-2875-11-294 PMC3502494

[B5] Ngomane L de Jager C Changes in malaria morbidity and mortality in Mpumalanga Province, South Africa (2001-2009): a retrospective study Malar J 2012 11 19 [http://www.malariajournal.com/content/11/1/19] 22239855 10.1186/1475-2875-11-19 PMC3292497

[B6] Govere J Durrheim D Coetzee M Hunt RH Malaria in Mpumalanga, South Africa, with special reference to the period 1987-1999 S Afr J Sci 2001 97 55 58

[B7] Sharp BL le Sueur D Malaria in South Africa–the past, the present and selected implications for the future S Afr Med J 1996 86 83 9 [http://www.ncbi.nlm.nih.gov/pubmed/8685790] 8685790

[B8] Sharp B Craig M Mnzava A Curtis B Maharaj R Kleinschmidt I Review of malaria in South Africa Tech. rep., Health Systems Trust, 2001

[B9] Blumberg L Frean J Malaria control in South Africa - challenges and successes 2007 [http://www.samj.org.za/index.php/samj/article/view/304] 18250936

[B10] Sharp BL Kleinschmidt I Streat E Maharaj R Barnes KI Durrheim DN Ridl FC Morris N Seocharan I Kunene S La-Grange JJP Mthembu JD Maartens F Martin CL Barreto A Seven years of regional malaria control collaboration - Mozambique, South Africa and Swaziland Am J Trop Med Hyg 2007 76 42 47 17255227 PMC3749812

[B11] Maharaj R Morris N Seocharan I Kruger P Moonasar D Mabuza A Raswiswi E Raman J The feasibility of malaria elimination in South Africa Malar J 2012 11 423 [http://www.malariajournal.com/content/11/1/423] 23253091 10.1186/1475-2875-11-423 PMC3573969

[B12] Silal SP Barnes KI Kok G Mabuza A Little F Exploring the seasonality of reported treated malaria cases in Mpumalanga, South Africa PloS One 2013 8 e76640 [http://www.plosone.org/article/info%3Adoi%2F10.1371%2Fjournal.pone.0076640;jsessionid=C561869F9C330805F7031175535AF8A8] 24204650 10.1371/journal.pone.0076640 PMC3812179

[B13] Lubombo spatial development initiative 2014 [http://www.malaria.org.za/lsdi/home.html] 10.1186/s12936-016-1453-9 PMC4983057 27520364

[B14] Murray JD Mathematical Biology, Volume 2 2002 Berlin Heidelberg: Springer Verlag [http://books.google.com/books?hl=en&lr=&id=XbCuqjePs0MC&pgis=1]

[B15] Mandal S Sarkar RR Sinha S Mathematical models of malaria–a review Malar J 2011 10 202 [http://www.malariajournal.com/content/10/1/202] 21777413 10.1186/1475-2875-10-202 PMC3162588

[B16] Craig M Snow R le Sueur D A climate-based distribution model of malaria transmission in Sub-Saharan Africa Parasitol Today 1999 15 105 111 [http://dx.doi.org/10.1016/S0169-4758(99)01396-4] 10322323 10.1016/s0169-4758(99)01396-4

[B17] Coleman M Coleman M Mabuza AM Kok G Coetzee M Durrheim DN Using the SaTScan method to detect local malaria clusters for guiding malaria control programmes Malar J 2009 8 68 [http://www.malariajournal.com/content/8/1/68] 19374738 10.1186/1475-2875-8-68 PMC2679049

[B18] Montosi E Manzoni S Porporato A Montanari A An ecohydrological model of malaria outbreaks Hydrol Earth Syst Sci 2012 16 2759 2769 [http://www.hydrol-earth-syst-sci.net/16/2759/2012/hess-16-2759-2012.html]

[B19] Koella JC Antia R Epidemiological models for the spread of anti-malarial resistance Malar J 2003 2 1 3 12643812 10.1186/1475-2875-2-3 PMC151679

[B20] Statistical release Mid-year population estimates Tech. rep., Statistics South Africa, 2011 [http://www.statssa.gov.za/publications/P0302/P03022011.pdf]

[B21] Mortality and causes of death in South Africa, 2010: findings from death notification Tech. rep., Statistics South Africa, Pretoria 2013 [http://www.statssa.gov.za/publications/p03093/p030932010.pdf]

[B22] White LJ Maude RJ Pongtavornpinyo W Saralamba S Aguas R Van Effelterre T Day NPJ White NJ The role of simple mathematical models in malaria elimination strategy design Malar J 2009 8 212 [http://www.malariajournal.com/content/8/1/212] 19747403 10.1186/1475-2875-8-212 PMC2754494

[B23] Jeffery GM Eyles DE Infectivity to mosquitoes of *Plasmodium Falciparum* as related to gametocyte density and duration of infection Am J Trop Med Hyg 1955 4 781 789 [http://www.ncbi.nlm.nih.gov/pubmed/13259002] 13259002 10.4269/ajtmh.1955.4.781

[B24] Miller MJ Observations on the natural history of malaria in the semi-resistant West African Trans R Soc Trop Med Hyg 1958 52 152 68 [http://www.ncbi.nlm.nih.gov/pubmed/13543904] 13543904 10.1016/0035-9203(58)90036-1

[B25] Eyles DE Young MD The duration of untreated or inadequately treated *Plasmodium Falciparum* infections in the human host J Natl Malar Soc 1951 10 327 336 [http://www.ncbi.nlm.nih.gov/pubmed/14908561] 14908561

[B26] Collins WE Jeffery GM A retrospective examination of sporozoite- and trophozoite-induced infections with *Plasmodium Falciparum*: development of parasitologic and clinical immunity during primary infection Am J Trop Med Hyg 1999 61 1 Suppl 4 19 [http://www.ncbi.nlm.nih.gov/pubmed/10432041] 10432041 10.4269/tropmed.1999.61-04

[B27] Chitnis N Hyman JM Cushing JM Determining important parameters in the spread of malaria through the sensitivity analysis of a mathematical model Bull Math Biol 2008 70 1272 96 [http://www.ncbi.nlm.nih.gov/pubmed/18293044] 18293044 10.1007/s11538-008-9299-0

[B28] Thomson D A Research Into the Production, Life and Death of Crescents in Malignant Tertian Malaria, in Treated and Untreated Cases, by an Enumerative Method; The Leucocytes in Malarial Fever: A Method of Diagnosing Malaria Long After it is Apparently Cured 1911 Liverpool: University Press

[B29] Makanga M Krudsood S The clinical efficacy of artemether/lumefantrine (Coartem) Malar J 2009 8 Suppl 1 S5 [http://www.malariajournal.com/content/8/S1/S5] 19818172 10.1186/1475-2875-8-S1-S5 PMC2760240

[B30] Castillo-Riquelme M McIntyre D Barnes K Household burden of malaria in South Africa and Mozambique: is there a catastrophic impact? Trop Med Int Health 2008 13 108 122 [http://www.ncbi.nlm.nih.gov/pubmed/18291009] 18291009 10.1111/j.1365-3156.2007.01979.x

[B31] Hlongwana KW Zitha A Mabuza AM Maharaj R Knowledge and practices towards malaria amongst residents of Bushbuckridge, Mpumalanga, South Africa Afr J Prim Health Care Fam Med 2011 3 9 [http://www.phcfm.org/index.php/phcfm/article/view/257]

[B32] Cleveland RB STL: A seasonal-trend decomposition procedure based on loess J Off Stat 1990 **6**. [http://uctsfx.hosted.exlibrisgroup.com/uct?sid=google&auinit=RB&aulast=Cleveland&atitle=STL:+A+seasonal-trend+decomposition+procedure+based+on+loess&title=Journal+of+official+statistics&volume=6&issue=1&date=1990&spage=3&issn=0282-423X]

[B33] Cohen JM Moonen B Snow RW Smith DL How absolute is zero? An evaluation of historical and current definitions of malaria elimination Malar J 2010 9 213 [http://www.pubmedcentral.nih.gov/articlerender.fcgi?artid=2983111&tool=pmcentrez&rendertype=abstract] 20649972 10.1186/1475-2875-9-213 PMC2983111

[B34] Global malaria control and elimination. Report of a technical review Tech. rep., World Health Organization, 2009 [http://www.who.int/malaria/publications/atoz/9789241596756/en/]

[B35] Maude RJ Socheat D Nguon C Saroth P Dara P Li G Song J Yeung S Dondorp AM Day NP White NJ White LJ Optimising strategies for *Plasmodium Falciparum* malaria elimination in Cambodia: primaquine, mass drug administration and artemisinin resistance PloS One 2012 7 e37166 [http://dx.plos.org/10.1371/journal.pone.0037166] 22662135 10.1371/journal.pone.0037166 PMC3360685

[B36] Phiri K Esan M van Hensbroek MB Khairallah C Faragher B ter Kuile FO Intermittent preventive therapy for malaria with monthly artemether–lumefantrine for the post-discharge management of severe anaemia in children aged 4–59 months in southern Malawi: a multicentre, randomised, placebo-controlled trial Lancet Infec Dis 2012 12 191 200 22172305 10.1016/S1473-3099(11)70320-6

[B37] Mabaso MLH Sharp B Lengeler C Historical review of malarial control in southern African with emphasis on the use of indoor residual house-spraying Trop Med Int Health 2004 9 846 56 [http://www.ncbi.nlm.nih.gov/pubmed/15303988] 15303988 10.1111/j.1365-3156.2004.01263.x

[B38] White NJ The role of anti-malarial drugs in eliminating malaria Malar J 2008 7 Suppl 1 S8 [http://www.malariajournal.com/content/7/S1/S8] 19091042 10.1186/1475-2875-7-S1-S8 PMC2604872

[B39] Gosling RD Okell L Mosha J Chandramohan D The role of antimalarial treatment in the elimination of malaria Clin Microbiol Infect 2011 17 1617 1623 [http://www.ncbi.nlm.nih.gov/pubmed/21951597] 21951597 10.1111/j.1469-0691.2011.03660.x

[B40] World Health Organization Guidelines on prevention of the reintroduction of malaria Tech 2007 [http://books.google.com/books?id=BoHPq6KqqqMC&pgis=1]

[B41] Tatem AJ Smith DL International population movements and regional *Plasmodium falciparum* malaria elimination strategies Proc Natl Acad Sci U S A 2010 107 12222 12227 [http://www.pnas.org/content/107/27/12222.short] 20566870 10.1073/pnas.1002971107 PMC2901446

