# Supplementary material for: Towards malaria elimination in Mpumalanga, South Africa: a population-level mathematical modelling approach
Source: Malar J. 2014 Aug 3;13:297. doi: 10.1186/1475-2875-13-297 (PMC4127654; doi:10.1186/1475-2875-13-297)
Supplement: Additional file 1 — Mathematical Model Description. Additional file containing model equations, description and parameter values. [file 1475-2875-13-297-S1.pdf]

Towards malaria elimination in Mpumalanga, South Africa: A population-level mathematical modelling approach

Additional File 1: Mathematical Model Description

Sheetal P Silal *[1], Francesca Little [1] , Karen I Barnes [2] and Lisa J White [3,4]

[1]Department of Statistical Sciences, University of Cape Town, Cape Town, South Africa
[2]Division of Clinical Pharmacology, Department of Medicine, University of Cape Town, Cape Town, South Africa
[3]Mahidol-Oxford Tropical Medicine Research Unit, Mahidol University, Bangkok, Thailand
[4]Centre for Tropical Medicine, Nuffield Department of Clinical Medicine, Churchill Hospital, University of Oxford, Oxford, UK

Email: Sheetal P Silal *- sheetal.silal@uct.ac.za; Francesca Little - francesca.little@uct.ac.za; Karen I Barnes - karen.barnes@uct.ac.za; Lisa J White - lisa@tropmedres.ac;

*Corresponding author

Base Model of Transmission

The model is a deterministic ordinary non-linear differential equation representation of the dynamics of the human population. In the model, the population is divided into 9 compartments: the susceptible population (S), the asexual blood stage only (B_l and B_f) for locally and imported infections respectively and the infectious gametocyte stage (I_l and I_f) for locally and imported infections respectively. The blood stage and infectious stage compartments are further stratified according to whether the infection is treated or not. The liver stage of the infection is incorporated as a delay in the flow between the Susceptible and Blood Stage compartments. As this is a low transmission environment, immunity and super-infection are rare and are excluded from this model. While the seasonal nature of transmission is incorporated in the model, the mosquito population is not modelled directly as it is assumed that the mosquito dynamics operate on a faster time-scale than the human dynamics and as such the mosquito population can be considered to be at equilibrium with respect to changes in the human population [1]. In the absence of sufficient data, asymptomatic infections and human migration are included indirectly in the model.The impact of the movement of infected individuals from external sources is captured through the parameter λ_f, the foreign force of infection i.e. the force of infection that generates imported infections. The impact of asymptomatic infections is captured by repeating the model analysis for a low assumption on the

proportion seeking treatment.

This system is described by a set of non-linear differential equations of form:

$$
\begin{aligned}
\frac{dS}{dt} &= \mu N - \lambda_l[t-\sigma_1]seas_l[t]S - \lambda_f[t-\sigma_1]seas_f[t]S + \frac{1}{r+\tau}(B_{l,tr} + I_{l,tr} + B_{f,tr} + I_{f,tr}) + \frac{1}{\delta}(I_{l,u} + I_{f,u}) - \mu S \\
\frac{dB_{l,tr}}{dt} &= p\lambda_l[t-\sigma_1]seas_l[t]S - \frac{1}{\sigma_2}B_{l,tr} - \frac{1}{r+\tau}B_{l,tr} - \mu B_{l,tr} \\
\frac{dI_{l,tr}}{dt} &= \frac{1}{\sigma_2}B_{l,tr} - \frac{1}{r+\tau}I_{l,tr} - \mu I_{l,tr} \\
\frac{dB_{l,u}}{dt} &= (1-p)\lambda_l[t-\sigma_1]seas_l[t]S - \frac{1}{\sigma_2}B_{l,u} - \mu B_{l,u} \\
\frac{dI_{l,u}}{dt} &= \frac{1}{\sigma_2}B_{l,u} - \frac{1}{\delta}I_{l,u} - \mu I_{l,u} \\
\frac{dB_{f,tr}}{dt} &= p\lambda_f[t-\sigma_1]seas_f[t]S - \frac{1}{\sigma_2}B_{f,tr} - \frac{1}{r+\tau}B_{f,tr} - \mu B_{f,tr} \\
\frac{dI_{f,tr}}{dt} &= \frac{1}{\sigma_2}B_{f,tr} - \frac{1}{r+\tau}I_{f,tr} - \mu I_{f,tr} \\
\frac{dB_{f,u}}{dt} &= (1-p)\lambda_f[t-\sigma_1]seas_f[t]S - \frac{1}{\sigma_2}B_{f,u} - \mu B_{f,u} \\
\frac{dI_{f,u}}{dt} &= \frac{1}{\sigma_2}B_{f,u} - \frac{1}{\delta}I_{f,u} - \mu I_{f,u}
\end{aligned}
$$

where

$$\lambda_l = (1 - vc[t])\beta_l \times \frac{I_{l,u} + I_{l,tr} + I_{f,u} + I_{f,tr}}{N}$$

and subscript l refers to locally sourced infections, f: foreign sourced infections, u: untreated infections and tr: treated infections. Thus local transmission is a function of the force of infection λ, the annual number of mosquito bites per person x proportion of bites testing positive for sporozoites (β), seasonality of transmission ($seas$) and vector control (vc). Data fitting produces an estimate for the scalar β and any modelled increases or decreases in the *level* of transmission are due to changes in vector control.

Transmission model with interventions

In this model the following interventions are modelled: Mass Drug Administration, Mass Screen and Treat, Scale-up of Vector Control and Foreign Source Reduction.

The following scenarios are tested:

(a) Scale up Vector Control so as to decrease β_l by a further (i) 10% and (ii) 20%

(b) Mass Drug Administration at 80% coverage over two months

  (i) annually over the peak of the season

  (ii) annually over the trough of the season

  (iii) six consecutive rounds over twelve months

(c) Mass Screen and Treat on imported infections at 70% coverage for six months over the malaria season

(d) (b-iii) & annual rounds of (c)

(e) (b-iii) & annual rounds of (c) & (a-i)

(f) Reducing the foreign source of infection by 70%

(g) (b-iii) & reducing the foreign source of infection by 100% (Hypothetical)

This system is described by a set of non-linear differential equations and is depicted in Figure 1:

$$\frac{dS}{dt} = \mu N - \lambda_l[t-\sigma_1]seas_l[t]S - \lambda_f[t-\sigma_1]seas_f[t](1-fsr[t])S + \frac{1}{r+\tau}(B_{l,tr}+I_{l,tr}+B_{f,tr}+I_{f,tr}) + \frac{1}{\delta}(I_{l,u}+I_{f,u}) - \frac{1}{mrate[t]}S + \frac{1}{ass[t]}S_{mda} + \frac{1}{pro_{MSAT}[t]}S_{msat} - \mu S$$

$$\frac{dB_{l,tr}}{dt} = p\lambda_l[t-\sigma_1]seas_l[t]S - \frac{1}{\sigma_2}B_{l,tr} - \frac{1}{r+\tau}B_{l,tr} - \frac{1}{mrate[t]}B_{l,tr} + \frac{1}{ass[t]}B_{mda,l,tr} - \mu B_{l,tr}$$

$$\frac{dI_{l,tr}}{dt} = \frac{1}{\sigma_2}B_{l,tr} - \frac{1}{r+\tau}I_{l,tr} - \frac{1}{mrate[t]}I_{l,tr} + \frac{1}{ass[t]}I_{mda,l,tr} - \mu I_{l,tr}$$

$$\frac{dB_{l,u}}{dt} = (1-p)\lambda_l[t-\sigma_1]seas_l[t]S - \frac{1}{\sigma_2}B_{l,u} - \frac{1}{mrate[t]}B_{l,u} + \frac{1}{ass[t]}B_{mda,l,u} - \mu B_{l,u}$$

$$\frac{dI_{l,u}}{dt} = \frac{1}{\sigma_2}B_{l,u} - \frac{1}{\delta}I_{l,u} - \frac{1}{mrate[t]}I_{l,u} + \frac{1}{ass[t]}I_{mda,l,u} - \mu I_{l,u}$$

$$\frac{dB_{f,tr}}{dt} = p(1-msprop[t])\lambda_f[t-\sigma_1]seas_f[t](1-fsr[t])S - \frac{1}{\sigma_2}B_{f,tr} - \frac{1}{r+\tau}B_{f,tr} - \frac{1}{mrate[t]}B_{f,tr} + \frac{1}{ass[t]}B_{mda,f,tr} - \mu B_{f,tr}$$

$$\frac{dI_{f,tr}}{dt} = \frac{1}{\sigma_2}B_{f,tr} - \frac{1}{r+\tau}I_{f,tr} - \frac{1}{mrate[t]}I_{f,tr} + \frac{1}{ass[t]}I_{mda,f,tr} - \mu I_{f,tr}$$

$$\frac{dB_{f,u}}{dt} = (1-p)(1-msprop[t])\lambda_f[t-\sigma_1]seas_f[t](1-fsr[t])S - \frac{1}{\sigma_2}B_{f,u} - \frac{1}{mrate[t]}B_{f,u} + \frac{1}{ass[t]}B_{mda,f,u} - \mu B_{f,u}$$

$$\frac{dI_{f,u}}{dt} = \frac{1}{\sigma_2}B_{f,u} - \frac{1}{\delta}I_{f,u} - \frac{1}{mrate[t]}I_{f,u} + \frac{1}{ass[t]}I_{mda,f,u} - \mu I_{f,u}$$

$$\frac{dS_{mda}}{dt} = \frac{1}{mrate[t]}S + adh * \frac{1}{pro_{MDA}[t]}(B_{mda}+I_{mda}) - \frac{1}{ass[t]}S_{mda} - \mu S_{mda}$$

$$\frac{dB_{mda}}{dt} = \frac{1}{mrate[t]}(B_{l,tr}+B_{l,u}+B_{f,tr}+B_{f,u}) - \frac{1}{\sigma_2}B_{mda} - adh * \frac{1}{pro_{MDA}[t]}B_{mda} - \mu B_{mda}$$

$$\frac{dI_{mda}}{dt} = \frac{1}{mrate[t]}(I_{l,tr}+I_{l,u}+I_{f,tr}+I_{f,u}) + \frac{1}{\sigma_2}B_{mda} - adh * \frac{1}{pro_{MDA}[t]}I_{mda} - \mu I_{mda}$$

$$\frac{dS_{msat}}{dt} = adh * \frac{1}{r}I_{msat} - \frac{1}{pro_{MSAT}[t]}S_{msat} - \mu S_{msat}$$

$$\frac{dI_{msat}}{dt} = msprop[t]\lambda_f[t-\sigma_1]seas_f[t](1-fsr[t])S - adh * \frac{1}{r}I_{msat} - \mu I_{msat}$$

$$\frac{dB_{mda,l,tr}}{dt} = p\lambda_l[t-\sigma_1]seas_l[t]S_{mda} - \frac{1}{\sigma_2}B_{mda,l,tr} - \frac{1}{r+\tau}B_{mda,l,tr} - \frac{1}{ass[t]}B_{mda,l,tr} - \mu B_{mda,l,tr}$$

$$\frac{dI_{mda,l,tr}}{dt} = \frac{1}{\sigma_2}B_{mda,l,tr} - \frac{1}{r+\tau}I_{mda,l,tr} - \frac{1}{ass[t]}I_{mda,l,tr} - \mu I_{mda,l,tr}$$

$$\frac{dB_{mda,l,u}}{dt} = (1-p)\lambda_l[t-\sigma_1]seas_l[t]S_{mda} - \frac{1}{\sigma_2}B_{mda,l,u} - \frac{1}{ass[t]}B_{mda,l,u} - \mu B_{mda,l,u}$$

$$\frac{dI_{mda,l,u}}{dt} = \frac{1}{\sigma_2}B_{mda,l,u} - \frac{1}{\delta}I_{mda,l,u} - \frac{1}{ass[t]}I_{mda,l,u} - \mu I_{mda,l,u}$$

$$\frac{dB_{mda,f,tr}}{dt} = p(1-msprop[t])\lambda_f[t-\sigma_1]seas_f[t](1-fsr[t])S_{mda} - \frac{1}{\sigma_2}B_{mda,f,tr} - \frac{1}{r+\tau}B_{mda,f,tr} - \frac{1}{ass[t]}B_{mda,f,tr} - \mu B_{mda,f,tr}$$

$$\frac{dI_{mda,f,tr}}{dt} = \frac{1}{\sigma_2}B_{mda,f,tr} - \frac{1}{r+\tau}I_{mda,f,tr} - \frac{1}{ass[t]}I_{mda,f,tr} - \mu I_{mda,f,tr}$$

$$\frac{dB_{mda,f,u}}{dt} = (1-p)(1-msprop[t])\lambda_f[t-\sigma_1]seas_f[t](1-fsr[t])S_{mda} - \frac{1}{\sigma_2}B_{mda,f,u} - \frac{1}{ass[t]}B_{mda,f,u} - \mu B_{mda,f,u}$$

$$\frac{dI_{mda,f,u}}{dt} = \frac{1}{\sigma_2}B_{mda,f,u} - \frac{1}{\delta}I_{mda,f,u} - \frac{1}{ass[t]}I_{mda,f,u} - \mu I_{mda,f,u}$$

where

$$\lambda_l = (1-vc[t]-vc_{add}[t])\,\beta_l \times \frac{I_{,l,u}+I_{l,tr}+I_{f,u}+I_{f,tr}+I_{mda}+I_{msat}+I_{mda,l,u}+I_{mda,l,tr}+I_{mda,f,u}+I_{mda,f,tr}}{N}$$

and subscript l refers to locally sourced infections, f: foreign sourced infections, u: untreated infections and tr: treated infections.

Tables

Table 1 - Compartment Descriptions table

Compartment	Description
S	Susceptible Population
$B_{l,tr}$	Population with **Blood** Stage **local** Infections that are **treated**
$I_{l,tr}$	Population with **Infectious** Stage **local** Infections that are **treated**
$B_{l,u}$	Population with **Blood** Stage **local** Infections that are **not treated**
$I_{l,u}$	Population with **Infectious** Stage **local** Infections that are **not treated**
$B_{f,tr}$	Population with **Blood** Stage **foreign** Infections that are **treated**
$I_{f,tr}$	Population with **Infectious** Stage **foreign** Infections that are **treated**
$B_{f,u}$	Population with **Blood** Stage **foreign** Infections that are **not treated**
$I_{f,u}$	Population with **Infectious** Stage **foreign** Infections that are **not treated**
S_{mda}	Susceptible Population having received MDA in a given cycle
B_{mda}	Population with **Blood** stage infections having received MDA in a given cycle
I_{mda}	Population with **Infectious** stage infections having received MDA in a given cycle
S_{msat}	Susceptible Population having received MSAT in a given cycle
I_{msat}	Population with **Blood** and **Infectious** stage infections having received MSAT in a given cycle
$B_{mda,l,tr}$	Population (infected after MDA) with **Blood** Stage **local** Infections that are **treated**
$I_{mda,l,tr}$	Population (infected after MDA) with **Infectious** Stage **local** Infections that are **treated**
$B_{mda,l,u}$	Population (infected after MDA) with **Blood** Stage **local** Infections that are **not treated**
$I_{mda,l,u}$	Population (infected after MDA) with **Infectious** Stage **local** Infections that are **not treated**
$B_{mda,f,tr}$	Population (infected after MDA) with **Blood** Stage **foreign** Infections that are **treated**
$I_{mda,f,tr}$	Population (infected after MDA) with **Infectious** Stage **foreign** Infections that are **treated**
$B_{mda,f,u}$	Population (infected after MDA) with **Blood** Stage **foreign** Infections that are **not treated**
$I_{mda,f,u}$	Population (infected after MDA) with **Infectious** Stage **foreign** Infections that are **not treated**

Table 2 - Full Parameter table

Table providing the values, descriptions and sources of the parameters driving the base and intervention mathematical models of transmission. In the intervention model, it is further assumed that Mass Drug

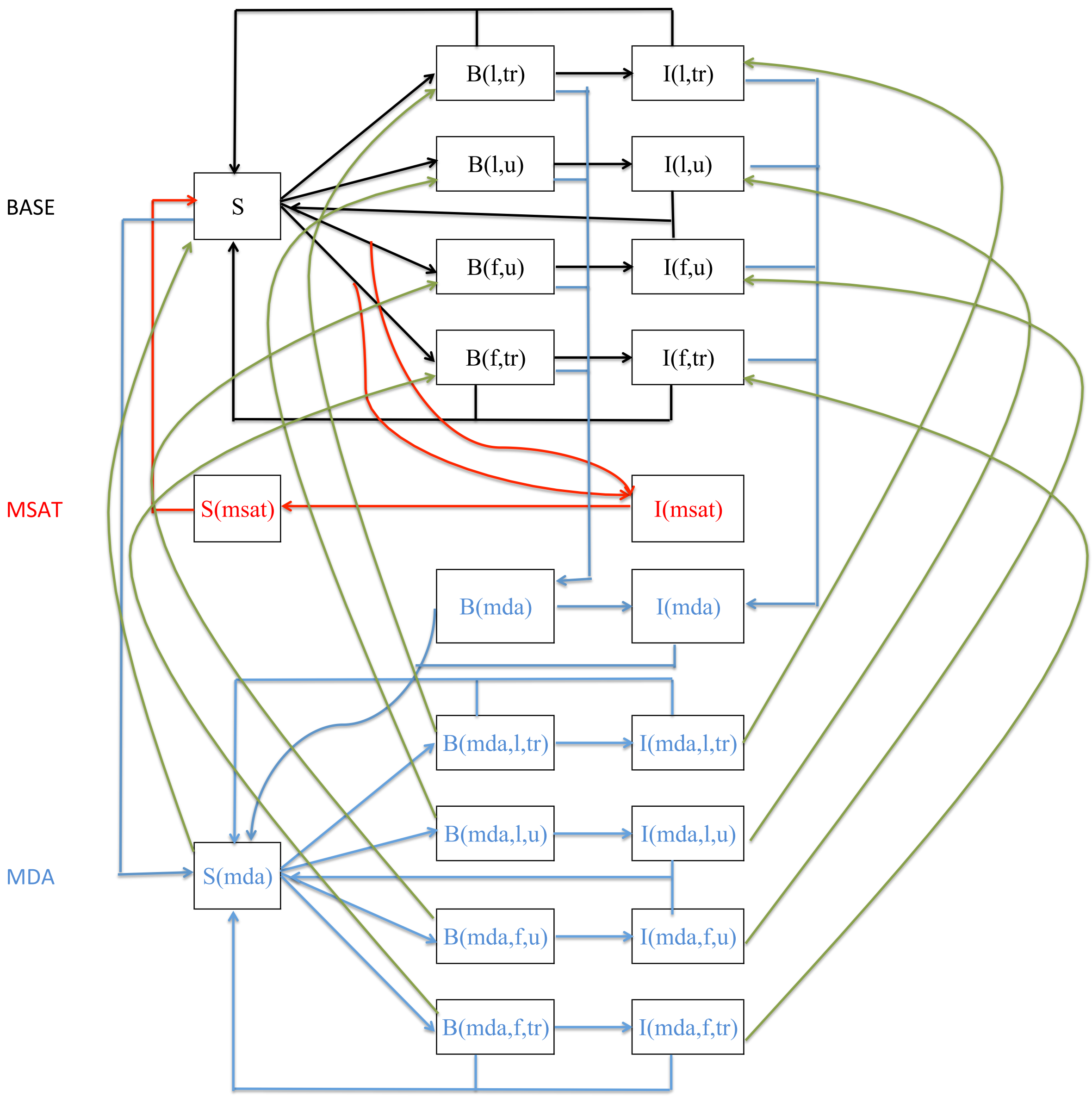

Figure 1: Model flow: Base Model (black) with interventions: MSAT(red), MDA(blue). The assimilation of the population having been subjected to MDA, but were infected beyond the prophylactic effect of the drug ,during the MDA cycle is represented in green.This is necessary as the duration of the MDA cycle is 8 weeks where as the prophlyactic period of the drug is only 4 weeks, so it is possible to get infected again within the 8 week period after being subjected to MDA. If these infections were accounted for in the base model compartments, it would be possible to receive MDA again, which is not usually the case.

administration is subjected only once to members of the population during a particular MDA cycle. While, a prophylactic effect of drug is accounted for, it is still possible to be infected after the protective period but before the MDA cycle is completed. Should this be the case, the proportion of the population will not receive MDA in that cycle again, but will be subject to routine treatment as per the base model. MSAT is applied to new imported infections (arising from a foreign source of infection λ_f) as they enter Mpumalanga from the foreign source and are assumed to be infectious.

Table 1: Full Parameter table

Parameter	Description	Value	Source
	**Base Model Parameters**		
N	Population size	4×10^6	[2]
μ	Mortality Rate	$\frac{105}{10000}$	[3]
δ	Natural recovery period	26 weeks	[4–6]
σ_1	Period between liver stage and blood stage	$\underline{7}$ days (5-10)	[7–9]
σ_2	Period between blood stage and onset of gametocytemia	1 week	[4,10]
r	AL elimination half-life	6 days	[11]
τ	Time to seek treatment	1/2 week	Expert opinion
p	Proportion that receive treatment	0.95	[12,13]
$seas_l$	Seasonal forcing function for locally sourced cases	Derived from data	[14]
$seas_f$	Seasonal forcing function for foreign sourced cases	Derived from data	[14]
β_l	Annual number of mosquito bites per person x proportion of bites testing positive for sporozoites	39.170 (38.894, 39.448)	Estimated from model fitting process
λ_f	Force of imported infections	0.002163 (0.002124, 0.002202)	Estimated from model fitting process
λ_l	Force of locally sourced infections	$(1 - vc[t])\beta_l \times \frac{I_{l,u}+I_{l,tr}+I_{f,u}+I_{f,tr}}{N}$	
	**Vector Control**		
$vc[t]$	$vccov \times vceff$		
$vccov$	Vector Control Coverage	0.22-0.90	Derived from data
$vceff$	Effectiveness of vector control	0.9060 (0.8884, 0.9212)	Estimated from model-fitting process
$vc_{add}[t]$	Additional Vector Control Coverage	vcaddon $\times$ vcadd	
$vcaddon$	Additional Vector Control Switch	Binary	
$vcadd$	Additional Vector Control Coverage	Scenario list	
	**Mass Drug Administration**		
$mrate[t]$	Rate of MDA Take-up	mdaon(-log(1-mcov)/mdur)	

$mdaon$	Mass Drug Administration Switch	Binary	
$mcov$	MDA coverage	80%	
$mdur$	Duration of MDA cycle	8 weeks	
$pro_{MDA}[t]$	Drug Protection period	4 weeks	[15]
adh	Adherence to Treatment	90%	[16]
$ass^{-1}[t]$	Rate of assimilation at the end of MDA cycle	Shift parameter	Modelling construct
**Mass Screen and Treat**			
$msprop[t]$	Proportion Screened and Treated through Border Control	mson × mscov	
$mson$	Mass Screen and Treat Switch	Binary	
$mscov$	MSAT coverage	70%	
$pro_{MSAT}[t]$	Drug Protection period	4 weeks	[15]
**Foreign Source Reduction**			
$fsr[t]$	Proportion reduced of the force of imported infections	fsron × fsrprop	
$fsron$	Foreign Source Reduction Switch	Binary	
$fsrprop[t]$	Proportional reduction	Scenario list	

Vector Control

Indoor Residual spraying is the primary vector control intervention employed in Mpumalanga. The data on the number of structures sprayed in Mpumalanga is provided by the Malaria Elimination Programme and has already been presented in Ngomane and de Jager (2012) and is depicted in Figure 2 [17]. Given that IRS is not 100% effective, a parameter on the effectiveness of IRS $vceff$ has been estimated in the data-fitting process.

Asymptomatic Infections

As asymptomatic infections are common even in low transmission areas, it is important to consider their impact in this model [18, 19]. In the absence of any data on the prevalence of asymptomatic infections in Mpumalanga, their impact has been assessed by re-running the analysis reducing the probability of treatment from 95% to 50%. By re-fitting the model to the data and re-running the analysis, the model predicts that it is only through action that reduces imported infections like extreme source reduction that elimination (as defined by the threshold used in the model) is possible (Figure 3). This is in line with the results predicted by the base model.

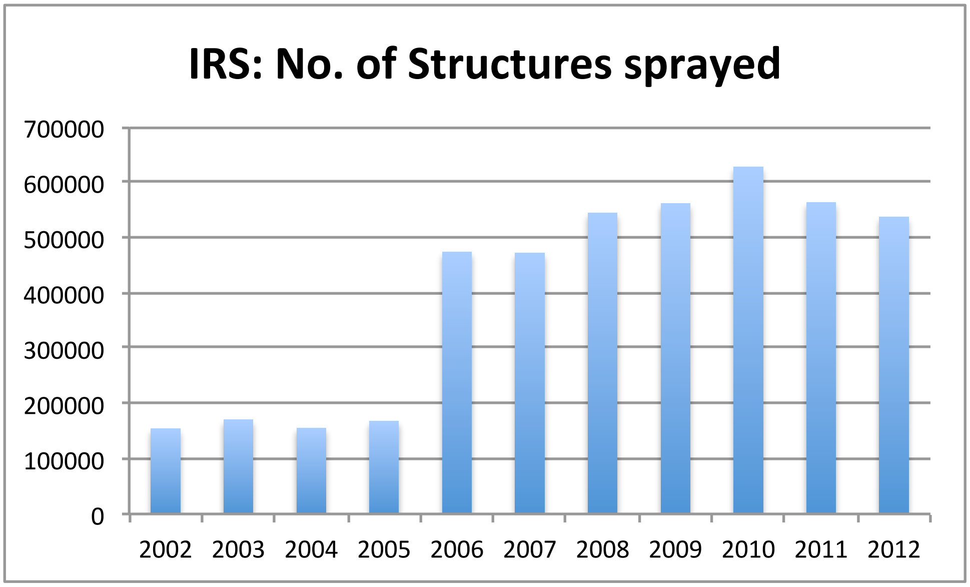

Figure 2: Number of structures sprayed in Mpumalanga between 2002 and 2012

Data Fitting Method

The model is fitted to weekly incidence data of treated cases from 2002 to 2008, and then validated with data from 2009 to 2012. The model is run from 1990 to reach a steady state before being fitted to data from 2002. IRS coverage and drug treatment are included in the model for the data fitting. The model output (local and imported treated cases) are fitted to the data using the maximum likelihood approach assuming an underlying Poisson distribution with canonical parameter λ as the average number of treated cases per week. The population-level non-linear differential equation model is expressed in terms of average rates of movement between compartments.

The Poisson probability of observing x counts when the average is λ is given by

$$P(x|\lambda) = \frac{\lambda^x \exp^{-\lambda}}{x!}.$$

As the model is being fitted to time series data with N time bins, λ, the expected number of counts per bin is a function of time. Assuming the independence of data in each time bin reduces the likelihood to

$$L(\lambda_i|x_i) = \prod_{i=1}^{N} \frac{\lambda_i^{x_i} \exp^{-\lambda_i}}{x_i!}$$

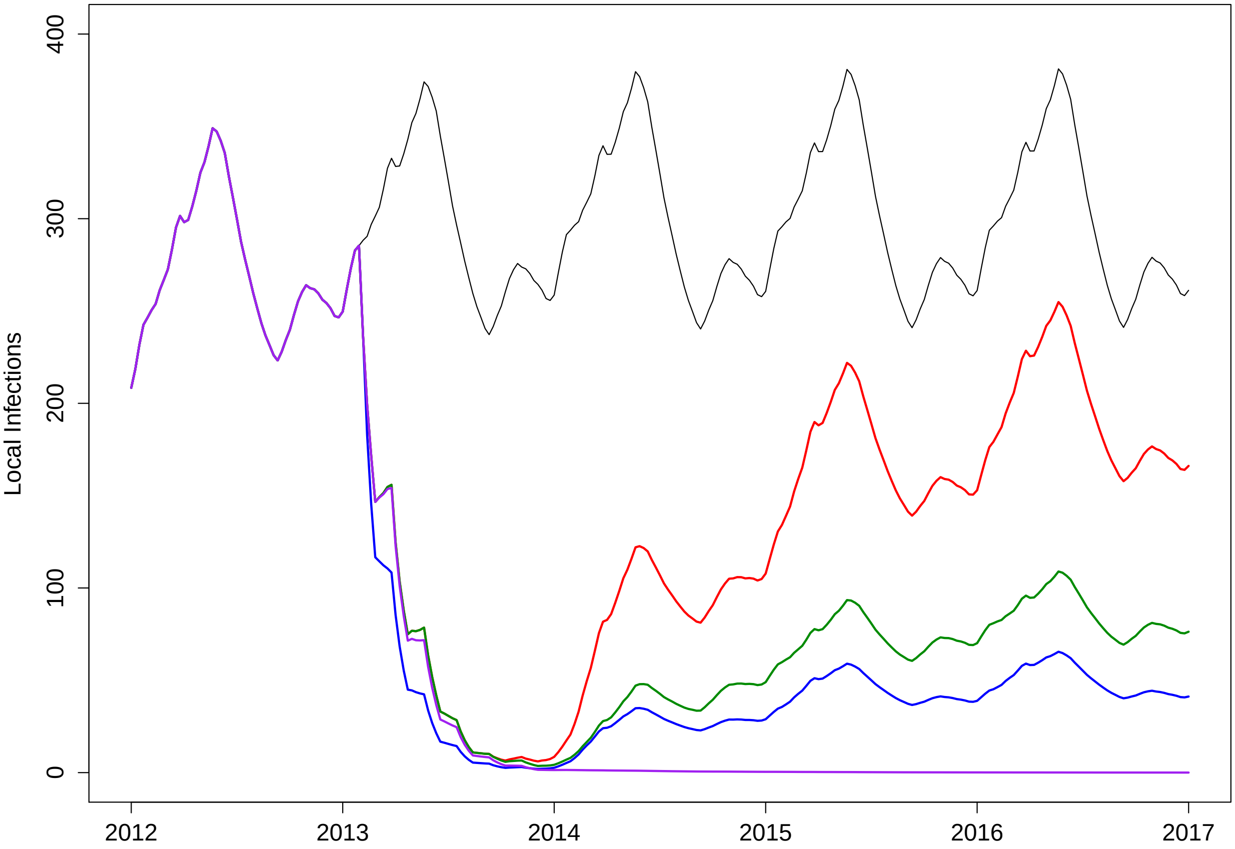

Figure 3: 50% probability of treatment: Predicted impact on local infections of combination of interventions on local infections: Black: No additional interventions, Red: 70% coverage of FSAT on local population with new imported infections following six consecutive two-monthly rounds of MDA at 80% coverage, Blue: same as red (MDA+FSAT) with increased vector control to decrease transmission by a further 20%, Green: six consecutive two-monthly rounds of MDA with increased vector control, and 70% decrease in the foreign force of infection, Purple: six consecutive two-monthly rounds of MDA with zero imported infections

and the log likelihood becomes

$$ln(L(\lambda_i|x_i)) = \sum_{i=1}^{N} x_i ln(\lambda_i) - \lambda_i - ln(x_i!).$$

The model output is fitted to two sets of data for each weekly time bin: locally sourced treated cases (l) and imported treated cases (f). Under the assumption of independence, the log likelihood to be maximised is

$$ln(L(\lambda_{l,i}\lambda_{f,i}|x_{l,i}, x_{f,i})) \propto \sum_{i=1}^{n} x_{l,i} ln(\lambda_{l,i}) - \lambda_{l,i} + x_{f,i} ln(\lambda_{f,i}) - \lambda_{f,i}$$

The log-likelihood is negated and minimised using the optim function implementing the Nelder and Mead algorithm in the R package Stats [20]. The parameters β_l and λ_f are estimated through this data fitting process. As the Nelder and Mead algorithm is a local search method, it is necessary to perform the optimisation from different starting points. The optimisation is performed 10000 times with starting values sampled from a Latin hypercube framework. The parameter estimates and their standard errors are retrieved from the optimisation output and are presented in Table 1. The model with the estimated parameter values is then run for a further 3 years (including IRS at comparative levels) to be further validated by comparison to data between 2009 and 2012.

References

1. Koella JC, Antia R: **Epidemiological models for the spread of anti-malarial resistance**. *Malar J* 2003, **2**.
2. **Statistical release Mid-year population estimates**. Tech. rep., Statistics South Africa 2011, [http://www.gov.za/publications/P0302/P03022011.pdf].
3. **Mortality and causes of death in South Africa, 2010: Findings from death notification**. Tech. rep., Statistics South Africa, Pretoria 2013, [http://www.gov.za/publications/p03093/p030932010.pdf].
4. Jeffery GM, Eyles DE: **Infectivity to mosquitoes of *Plasmodium Falciparum* as related to gametocyte density and duration of infection.** *Am J Trop Med Hyg* 1955, **4**:781–789, [http://www.ncbi.nlm.nih.gov/pubmed/13259002].
5. Miller MJ: **Observations on the natural history of malaria in the semi-resistant West African.** *Trans R Soc Trop Med Hyg* 1958, **52**:152–68, [http://www.ncbi.nlm.nih.gov/pubmed/13543904].
6. White LJ, Maude RJ, Pongtavornpinyo W, Saralamba S, Aguas R, Van Effelterre T, Day NPJ, White NJ: **The role of simple mathematical models in malaria elimination strategy design.** *Malar J* 2009, **8**:212, [http://www.malariajournal.com/content/8/1/212].
7. Eyles DE, Young MD: **The duration of untreated or inadequately treated *Plasmodium Falciparum* infections in the human host.** *J Natl Malar Soc* 1951, **10**:327–336, [http://www.ncbi.nlm.nih.gov/pubmed/14908561].
8. Collins WE, Jeffery GM: **A retrospective examination of sporozoite- and trophozoite-induced infections with *Plasmodium Falciparum*: development of parasitologic and clinical immunity during primary infection.** *Am J Trop Med Hyg* 1999, **61**(1 Suppl):4–19, [http://www.ncbi.nlm.nih.gov/pubmed/10432041].
9. Chitnis N, Hyman JM, Cushing JM: **Determining important parameters in the spread of malaria through the sensitivity analysis of a mathematical model.** *Bull Math Biol* 2008, **70**:1272–96, [http://www.ncbi.nlm.nih.gov/pubmed/18293044].
10. Thomson D: *A Research Into the Production, Life and Death of Crescents in Malignant Tertian Malaria, in Treated and Untreated Cases, by an Enumerative Method; The Leucocytes in Malarial Fever: A Method of Diagnosing Malaria Long After it is Apparently Cured.* University Press 1911.
11. Makanga M, Krudsood S: **The clinical efficacy of artemether/lumefantrine (Coartem).** *Malar J* 2009, **8 Suppl 1**:S5, [http://www.malariajournal.com/content/8/S1/S5].
12. Castillo-Riquelme M, McIntyre D, Barnes K: **Household burden of malaria in South Africa and Mozambique: is there a catastrophic impact?** *Trop Med Int Health* 2008, **13**:108–122, [http://www.ncbi.nlm.nih.gov/pubmed/18291009].
13. Hlongwana KW, Zitha A, Mabuza AM, Maharaj R: **Knowledge and practices towards malaria amongst residents of Bushbuckridge, Mpumalanga, South Africa**. *Afr J Prim Health Care Fam Med* 2011, **3**:9 pages, [http://www.phcfm.org/index.php/phcfm/article/view/257/280].

14. Silal SP, Barnes KI, Kok G, Mabuza A, Little F: **Exploring the seasonality of reported treated malaria cases in Mpumalanga, South Africa.** *PloS One* 2013, **8**:e76640, [http://www.plosone.org/article/info\%3Adoi\%2F10.1371\%2Fjournal.pone.0076640;jsessionid=C561869F9C330805F7031175535AF8A8].
15. Phiri K, Esan M, van Hensbroek MB, Khairallah C, Faragher B, ter Kuile FO: **Intermittent preventive therapy for malaria with monthly artemether–lumefantrine for the post-discharge management of severe anaemia in children aged 4–59 months in southern Malawi: a multicentre, randomised, placebo-controlled trial**. *Lancet Infec Dis* 2012, **12**:191–200.
16. Barnes KI, Durrheim DN, Little F, Jackson A, Mehta U, Allen E, Dlamini SS, Tsoka J, Bredenkamp B, Mthembu DJ, White NJ, Sharp BL: **Effect of artemether-lumefantrine policy and improved vector control on malaria burden in KwaZulu-Natal, South Africa.** *PloS Med* 2005, **2**:e330, [http://dx.plos.org/10.1371/journal.pmed.0020330].
17. Ngomane L, de Jager C: **Changes in malaria morbidity and mortality in Mpumalanga Province, South Africa (2001- 2009): a retrospective study**. *Malar J* 2012, **11**:19, [http://www.malariajournal.com/content/11/1/19].
18. Harris I, Sharrock WW, Bain LM, Gray KA, Bobogare A, Boaz L, Lilley K, Krause D, Vallely A, Johnson ML, Gatton ML, Shanks GD, Cheng Q: **A large proportion of asymptomatic Plasmodium infections with low and sub-microscopic parasite densities in the low transmission setting of Temotu Province, Solomon Islands: challenges for malaria diagnostics in an elimination setting.** *Malar J* 2010, **9**:254, [http://espace.library.uq.edu.au/view/UQ:218971].
19. Okell LC, Bousema T, Griffin JT, Ouédraogo AL, Ghani AC, Drakeley CJ: **Factors determining the occurrence of submicroscopic malaria infections and their relevance for control.** *Nature communications* 2012, **3**:1237, [http://dx.doi.org/10.1038/ncomms2241].
20. Nelder JA, Mead R: **A Simplex Method for Function Minimization**. *The Computer Journal* 1965, **7**:308–313, [http://comjnl.oxfordjournals.org/content/7/4/308.abstract].
